# The relationship between anxiety and depression with smartphone addiction among college students: The mediating effect of executive dysfunction

**DOI:** 10.3389/fpsyg.2022.1033304

**Published:** 2023-01-11

**Authors:** JiaMin Ge, Ya Liu, Wenjing Cao, Shuyin Zhou

**Affiliations:** ^1^School of Educational Sciences, Chongqing Normal University, Chongqing, China; ^2^Key Laboratory of Applied Psychology, Chongqing Normal University, Chongqing, China

**Keywords:** anxiety, smartphone addiction, depression, executive function, I-PACE model, CIUT model

## Abstract

Smartphone addiction symptom is increasing globally. Many studies have found that negative emotion is associated with smartphone addiction, but few explore the mediating effect of executive dysfunction. In a large-scale, cross-sectional survey, 421 Chinese college students completed measures on anxiety, depression, smartphone addiction, and executive dysfunction. We surveyed the prevalence of depression, impaired executive function, and smartphone addiction. A confirmatory factor analysis was performed on the questionnaire structure, and the mediation models were used to examine the relationship between anxiety, depression, impaired executive function, and smartphone addiction. The main finding indicated that anxiety, depression, and executive dysfunction were positively and significantly associated with smartphone addiction. Executive dysfunction plays a mediation role between anxiety and depression with smartphone addiction. Specifically, executive dysfunction completely mediates the pathway of anxiety and smartphone addiction and partly mediates the path of depression and smartphone addiction. Depression directly predicted smartphone addiction positively but anxiety did not. The sample consisted of Chinese college students, which limits generalizability and self-reported lack of objectivity. The result suggests that we should pay more attention to the mediating role of executive dysfunction between negative emotion and smartphone addiction.

## 1. Introduction

According to 46% of smartphone users, they “could not live without their smartphones” (Smith, 2015). By June 2022, the number of smartphone network users in China had reached 1.047 billion, which is ~74.1% of the Chinese population (China Internet Information Center, 2022). By May 2022, 93~97% of U.S. adults had smartphones, up from 35% in 2013 (Statista, 2022). While the smartphone offers several benefits (Bertschek and Niebel, 2016; Lee et al., 2017), it also has plenty of negative consequences (Lin et al., 2014). For example, excessive smartphone use can lead to neck, shoulder, and low back pain (Hakala et al., 2006) as well as hearing and vision problems (Meo and Al-Drees, 2005). People even use the smartphone when driving, regardless of the relevant ban and danger (Caird et al., 2014). With increasing harmful effects, the new term “smartphone addiction” emerged (Lin et al., 2014).

Smartphone addiction is defined as “a new type of behavioral addiction caused by over-dependence and abuse of smartphones, resulting in psychological and behavioral problems” (Kwon et al., 2013b; Lee et al., 2014; Lin et al., 2014). A volume of research indicates that smartphone addiction is related to negative influences that have penetrated various aspects of life, such as musculoskeletal pain (Xie et al., 2016; Salvi and Battin, 2018), poor sleep quality (Li et al., 2020; Mei et al., 2022), loneliness (Liu et al., 2019), decreased life satisfaction (Lepp et al., 2014), interpersonal problems (Dwyer et al., 2018; Nayak, 2018), and poor academic performance (Yang Z. et al., 2019; Sapci et al., 2021). To the best of our knowledge, despite a vast array of negative consequences, the smartphone addiction rate is still rising. Recently, Olson et al. (2022) conducted a meta-analysis of studies published between 2014 and 2020 and discovered that the prevalence of smartphone addiction is increasing globally. Meanwhile, the highest smartphone addiction levels were in China and Saudi Arabia, where 36.6% of college students are particularly susceptible to smartphone addiction (Mei et al., 2022). Their daily average screen time has risen dramatically from 3.75 h in 2012 to 5.78 h in 2017 (Kim et al., 2019). Thus, it is necessary to investigate smartphone addiction formation among Chinese college students.

To capture the formational mechanism of smartphone addiction, we used a battery of theories and models, including the compensatory Internet use theory (CIUT) (Kardefelt-Winther, 2014), the Integrative Pathways Model (IPM) (Billieux et al., 2015), and the interaction of person-affect-cognition-execution model (I-PACE) (Brand et al., 2016, 2019). Although CIUT and I-PACE were initially used to describe Internet use disorder, smartphone addiction shares many common features with this disorder (Kwon et al., 2013a; Lin et al., 2014). It is even considered a special type of Internet addiction (Montag et al., 2021). In terms of CIUT, smartphone addiction is an unhealthy coping way to escape real life and adverse emotions and obtain emotional compensation in the virtual world. IPM offers three pathways to account for smartphone addiction. One of them, the path of excessive reassurance-seeking, indicates that psychologically vulnerable individuals keep using their smartphones to maintain relationships and seek reassurance from others. I-PACE emphasizes the interaction of the person, affective and cognitive responses to external or internal stimuli, and executive functions. Personal factors increase the risk of smartphone addiction, such as biopsychological constitution, psychopathological features (e.g., depression and anxiety), and personality. Affective and cognitive responses and executive function are mechanistic variables between the personal factor and smartphone addiction.

Mounting studies yielded insights into the relationship between negative emotions and smartphone addiction, especially anxiety and depression (Elhai et al., 2017a; Yang J. et al., 2019; Li et al., 2020). Additionally, the notion that anxiety and depression lead to smartphone addiction is consistent with CIUT, IPM, and I-PACE. Anxiety and depression give rise to smartphone addiction, a coping strategy to seek pleasure. Furthermore, some researchers explored the mediation mechanism underlying the relationship between anxiety and depression with smartphone addiction, for instance, boredom proneness (Wolniewicz et al., 2019; Wang et al., 2020), rumination (Elhai et al., 2018; Wang et al., 2020; Vally et al., 2021), and FoMO (fear of miss out) (Elhai et al., 2019, 2020; Vally et al., 2021). However, few empirical studies explored the mediation role of executive function between them, which is an essential component of the development of addiction behavior (Brand et al., 2016; Dominguez-Salas et al., 2016).

Executive function is a series of interactive higher cognitive functions whose core components are inhibitory control, cognitive flexibility, and working memory (Miyake et al., 2000). Negative affect is involved at the executive dysfunction level. On the one hand, existing studies demonstrate that anxiety impairs executive function (Darke, 1988; Derakshan et al., 2009; Ansari and Derakshan, 2010; Shields et al., 2016). According to the attention control theory (ACT) (Eysenck et al., 2007), anxiety occupies cognitive resources and leads to executive dysfunction. On the other hand, depression and cognitive disorders often coexist [American Psychiatric Association (APA), 2013]. A meta-analysis investigated the performance of depressed individuals using the n-back task and found significant working memory deficits (Nikolin et al., 2021). Another study found that antidepressants can improve cognitive function among depressed individuals (Prado et al., 2018).

In addition, executive dysfunction is not only a result variable of negative affect but also a robust predictor of addiction behavior (Brand et al., 2016). As expected, existing empirical studies also indicate that executive dysfunction and smartphone addiction are intercorrelated (Hadlington, 2015; Chen et al., 2016; Gao et al., 2020). Wilmer et al. (2017) also discussed the relationship between mobile technology habits and cognitive function in a review and considered it as nonsignificant.

In terms of our study variables integrated with I-PACE, anxiety and depression were conceptualized as personal factors and psychopathological features. Executive function was a crucial part of the model and a mediator between personal factors and addictive behavior. Addiction behavior is the outcome variable in the I-PACE. We focus on the specific addiction behavior, i.e., smartphone addiction. CIUT, IPM, and ACT also provide theoretical foundation for the hypothesized model. Given the theory and empirical evidence presented above, we speculate that executive dysfunction may play a mediation role in the relationship between anxiety and depression with smartphone addiction.

Thus, the objective of the present study was to examine, first, associations between anxiety, depression, and smartphone addiction, and, second, the mediation of executive functions between them. The hypotheses are as follows:

Hypothesis 1. Anxiety (*H1a*) and depression (*H1b*) are positively related to smartphone addiction.
Hypothesis 2. Anxiety (*H2a*) and depression (*H2b*) are positively related to executive dysfunction.
Hypothesis 3. Executive dysfunction is positively related to smartphone addiction.
Hypothesis 4. Anxiety and depression affect smartphone addiction through executive dysfunction.

**Figure 1:**
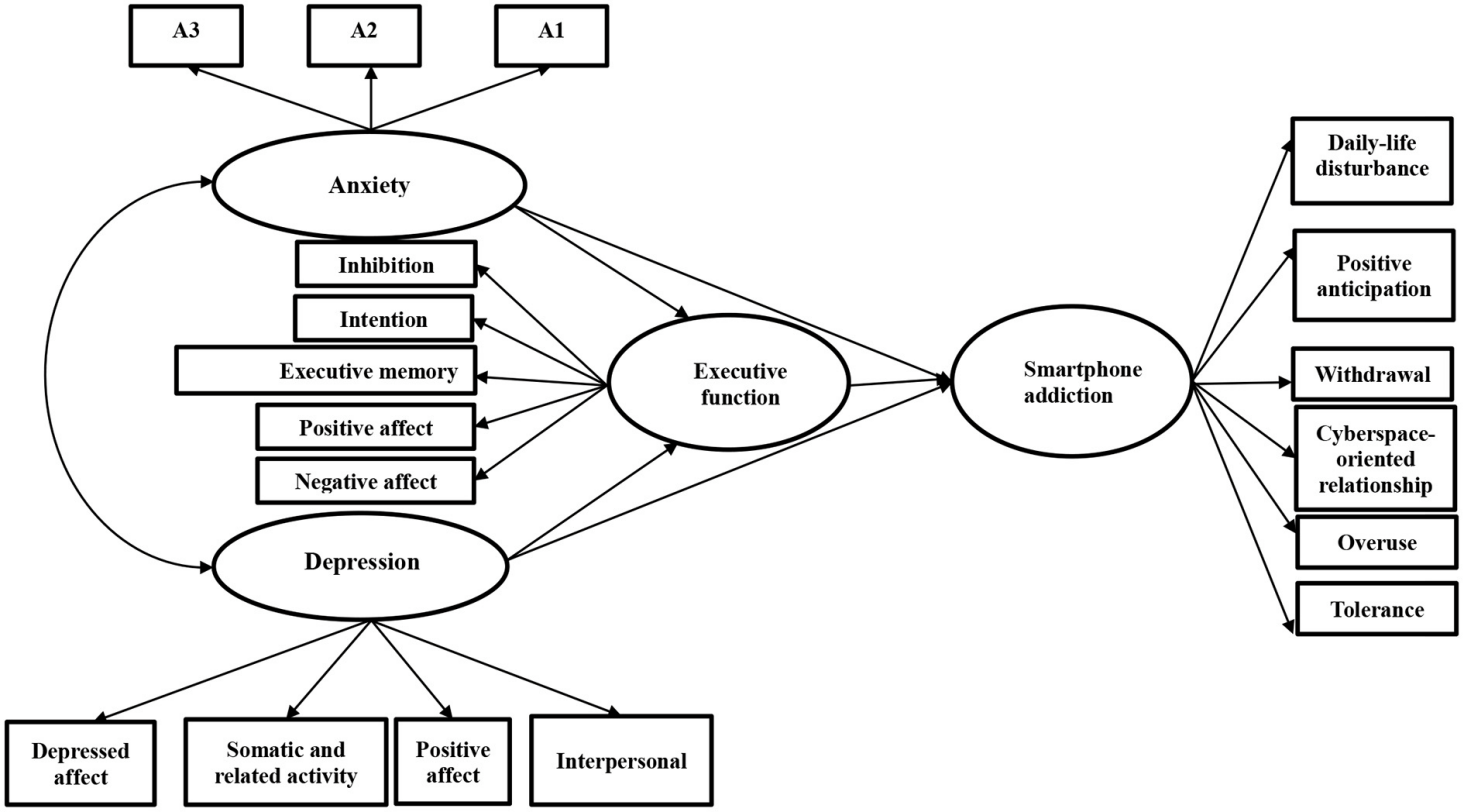
Hypothetical model concerning the mediation effect of executive dysfunction in the relationships between anxiety and depression with smartphone addiction.

The hypothetical model is shown in Figure 1.

The study has some meaningful contributions. First, we tested the validity of CIUT, IPM, and I-PACE. Second, we explored the executive function as the mediator between anxiety and depression with smartphone addiction, filling the research gap. Finally, the finding may provide valuable and complementary insights into smartphone treatment and help find the underlying reason for smartphone addiction formation to act appropriately.

## 2. Materials and methods

### 2.1. Participants

The participants were recruited from Chongqing Normal University. Out of 450 questionnaires, 29 invalid questionnaires were excluded. The response rate was 93.6%. A total of 421 college students participated in our survey. The sample size met the requirement put forward by James (2016). The mean age of the participants was 19.29 years (SD = 1.85, range =16–24 years). Of the participants, 290 students were female students (68.9%) and 131 were male students (31.1%). In addition, 168 students were from the countryside (39.9%), 67 students were from the township (15.9%), and 186 students were from the city (44.2%). They all completed the questionnaire measuring anxiety, depression, executive function, and smartphone addiction. The local research ethics committee of Chongqing Normal University approved this study, and all participants signed informed consent.

### 2.2. Measures

#### 2.2.1. Smartphone addiction

Smartphone addiction was measured using the smartphone addiction scale (SAS; Kwon et al., 2013b), consisting of 33 items. It includes the following six components: daily-life disturbance, positive anticipation, withdrawal, cyberspace-oriented relationship, overuse, and tolerance. The participant rated each item on a 6-point scale ranging from 1, strongly disagree, to 6, strongly agree, with higher scores indicating more smartphone addiction. The Cronbach's α of the SAS was 0.943.

The short version of the smartphone addiction scale (SAS-SV; Kwon et al., 2013a) is included in the full version and consists of 10 items. Like the full version, it adapts a Likert-type scale. According to SAS-SV, the cutoff value is 31 for boys and 33 for girls. The Cronbach's α of the SAS-SV was 0.877.

#### 2.2.2. Anxiety

Anxiety was measured using the trait version of the State-Trait Anxiety Inventory (STAI-T) (Spielberger, 1983) consisting of 20 items. Each item was rated on a 4-point scale ranging from 1, not at all to 4, always, with higher scores indicating higher anxiety levels. In the current study, Cronbach's α of the STAI-T was 0.878.

#### 2.2.3. Depression

Depression was measured using the Center for Epidemiological Studies Depression Scale (CES-D, Radloff, 1977), consisting of 20 items. Respondents indicate how often within the last week they experienced the symptoms on a 4-point scale (1 = “rarely or none of the time”; 2 = “some or little of the time”; 3 = “occasionally or a moderate amount of time”; 4 = “most or all of the time”). The scores for the 20 items are added, with higher scores representing worse conditions of depression. According to a meta-analysis of 23 studies (Vilagut et al., 2016), point 20 is more appropriate as the cutoff point than 16 (Radloff, 1977). The Cronbach's α for the CES-D was 0.881.

#### 2.2.4. Executive dysfunction

Executive dysfunction was measured by the Dysexecutive Questionnaire (DEX; Chan, 2001) consisting of 20 items. It includes the following five components: inhibition, intention, executive memory, positive affect, and negative affect. Each item was rated on a 5-point scale ranging from 0, never to 4, often. A higher score indicates a more impaired executive function. A DEX total score of ≥20 implies mild executive dysfunction, ≥28 implies moderate executive dysfunction, and ≥36 implies strong executive dysfunction (Bodenburg and Dopslaff, 2008). In this study, Cronbach's α of the DEX was 0.919.

### 2.3. Procedure

Students were invited to answer a questionnaire that included anxiety, depression, executive dysfunction, and smartphone addiction, which could be completed in ~15 min. All students received the same test and instructions.

### 2.4. Analysis

This study adapted SPSS 15.0 and Amos 26.0 software for the data analysis. First, confirmatory factor analysis (CFA) was used to examine whether the items within a construct are valid. Then, we performed a descriptive statistical analysis to identify the distribution of all variables and Pearson's correlation analysis to estimate the correlation coefficients between all variables. Subsequently, we conducted a Harman single-factor test to test possible common method biases. Finally, we examined the mediation role of executive dysfunction using the structural equation model.

## 3. Results

### 3.1. Preliminary analyses

The CFA was used to ensure the construct validity of the questionnaire. To the best of our knowledge, a model index fit of χ^2^/df <3, RMSEA < 0.08, SRMR < 0.10, and TLI and CFI > 0.90 is acceptable (Hu and Bentler, 1999). The preliminary analysis results revealed that there was an apparent four-factor structure for this questionnaire: χ^2^/df = 2.94, CFI = 0.95, NFI = 0.92, IFI = 0.95, RMSEA = 0.07, and SRMR = 0.05.

The descriptive statistical analysis of 421 college students is presented in Table 1. Correlation analysis showed that all self-report measures were significantly positively correlated.

**Table 1:** The correlation analysis between study variables.

	**M**	**SD**	**1**	**2**	**3**	**4**
1. Anxiety	43.57	8.99	1			
2. Depression	17.54	9.11	0.70^**^	1		
3. Executive dysfunction	23.86	12.46	0.56^**^	0.59^**^	1	
4. Smartphone addiction	102.61	27.34	0.36^**^	0.41^**^	0.46^**^	1

** *P* < 0.01.

We identified the mental health of college students based on the aforementioned cutoff scores of measurement tools. There are 46.3% of college students with smartphone addiction (SAS-SV, female students > 33, male students > 31) (Kwon et al., 2013a), 39.4% of them with depression (CES-D ≥ 20) (Vilagut et al., 2016), and 59.6% of them with executive dysfunction (DEX ≥ 20) (Bodenburg and Dopslaff, 2008). With attention to detail, there are 24.0% of college students with mild executive dysfunction (28 > DEX ≥ 20), 18.3% of them with moderate executive dysfunction (36 > DEX ≥ 28), and 17.3% of them with strong executive dysfunction (DEX ≥ 36).

### 3.2. Common method biases test

The Harman single-factor test was used to test possible common method biases (Podsakoff et al., 2003) of the collected data. The unrotated exploratory factor analysis results extracted a total of 19 factors with an eigenvalue >1, and the maximal items load onto a single factor was 23.29%, far <40%. Therefore, there was no serious common method bias in the data of this study.

### 3.3. Structural equation modeling

**Figure 2:**
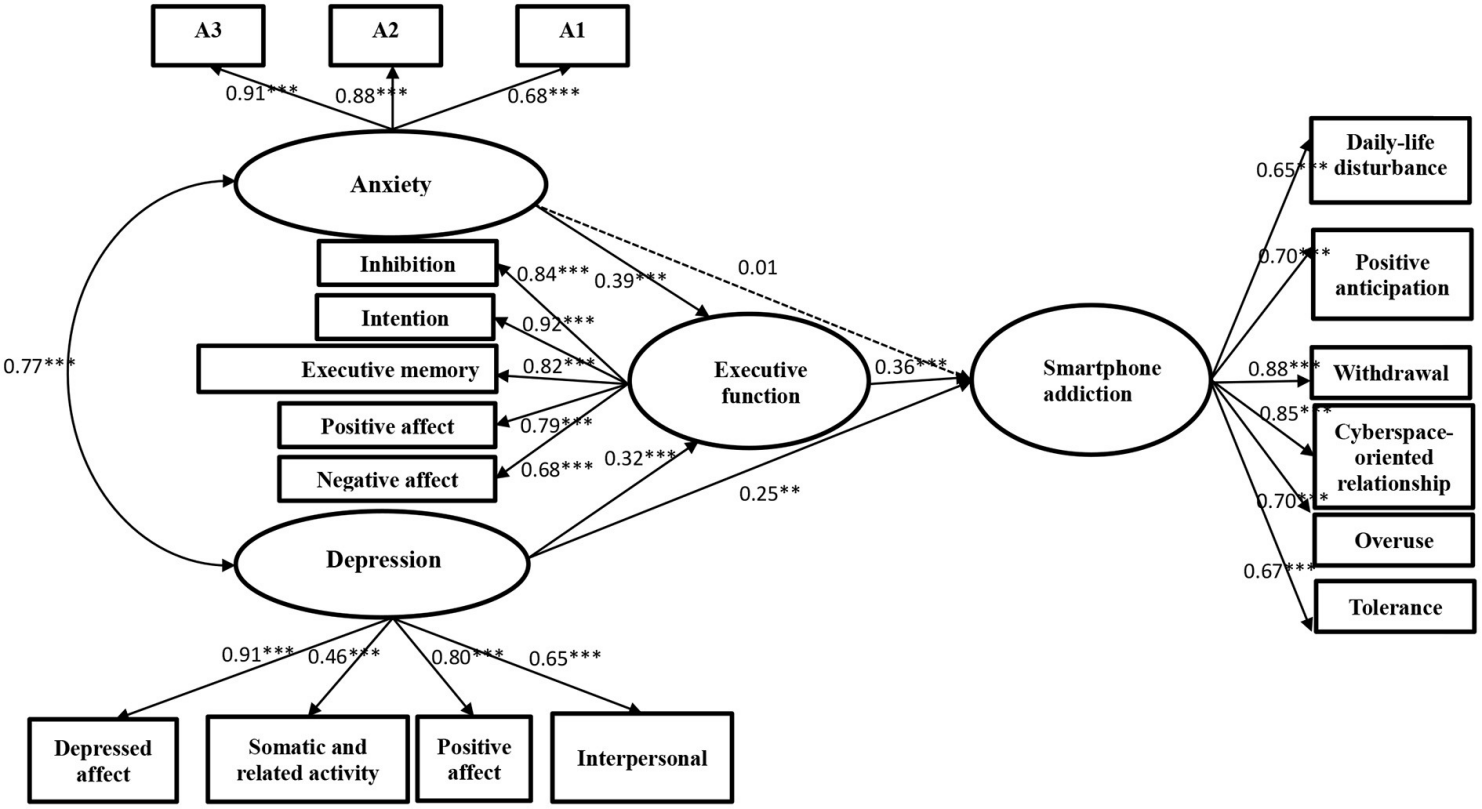
Final model concerning the mediation effect of executive dysfunction in the relationships between anxiety and depression with smartphone addiction. *P < 0.05, **P < 0.01, ***P < 0.001.

For path analysis, anxiety and depression were employed as the predictor variables, smartphone addiction as the outcome variable, and executive dysfunction as the mediating variable based on the hypothetical model. The result is shown in Figure 2. The fit indices are as follows: χ^2^/df = 2.97, CFI = 0.94, NFI = 0.92, IFI = 0.95, RMSEA = 0.07, and SRMR = 0.05. The fitness statistics are within a reasonable range (Hu and Bentler, 1999).

From the perspective of the model path, depression directly predicted smartphone addiction (r = 0.25, *P* = 0.007), but anxiety did not (r = 0.01, *P* = 0.92). In the mediation test of executive dysfunction, in one path, anxiety predicted executive dysfunction (r = 0.39, *P* < 0.001), and executive dysfunction predicted smartphone addiction (r = 0.36, *P* < 0.001), whereas in another path, depression predicted executive dysfunction (r = 0.32, *P* < 0.001). Using bias-corrected percentile bootstrap to test the ratio of the mediating effect to the total effect size, indirect path 1 (anxiety → executive dysfunction → smartphone addiction) was 28.2%, and the 95% interval was [0.25, 0.80]. Therefore, executive dysfunction is the complete intermediary variable of anxiety and smartphone addiction. Indirect path 2 (depression → executive dysfunction → smartphone addiction) was 22.4%, and the 95% interval was [0.14, 0.72]. Therefore, executive dysfunction is the part intermediary variable of depression and smartphone addiction.

## 4. Discussion

The hypotheses have been verified in the present study. We mainly found that negative emotion influences smartphone addiction through the mediator role of executive dysfunction, which is shown in two pathways. Executive dysfunction fully mediated the relationship between anxiety and smartphone addiction but partially mediated the relationship between depression and smartphone addiction.

The theoretical importance of the study results is two-fold. First, the results provide support for their application in terms of CIUT, IPM, and I-PACE. Second, we investigated the relationships between depression and anxiety with smartphone addiction and better understood the process of smartphone addiction development. Specifically, all hypotheses above were supported.

The results verified CIUT, IPM, and I-PACE once again (Elhai et al., 2019; Wang et al., 2020). According to CIUT, the motivation for Internet use is based on unmet real-life needs or psychological imbalance. In our study, individuals with anxiety and depression may suffer negative emotions in the real world, which leads to compensatory behavior, causing them to seek relief in the virtual world and increasing the possibility of smartphone addiction. Meanwhile, as explained by the excessive reassurance pathway of IPM, smartphone addiction is driven by maintaining the relationship and seeking reassurance. Individuals with anxiety or depression are prone to have this tendency. In addition, in terms of I-PACE, personal factors (e.g., anxiety and depression) may lead to addictive behavior. Poor executive function will decrease the ability to make wise decisions, promoting the development of addiction behavior. Executive dysfunction is a mechanistic variable that accounts for relationships between personal factors and smartphone addiction. On the whole, CIUT, IPM, and I-PACE can advance a reasonable understanding of smartphone addiction.

We explored the mechanisms of addiction formation through four hypotheses. As predicted by H1, depression and anxiety were associated with smartphone addiction. The results have been consistently demonstrated in many studies (Elhai et al., 2017a; Yang J. et al., 2019; Li et al., 2020). Our results are consistent with CIUT, IPM, and I-PACE, suggesting that individuals with negative emotions or existing psychopathological features may react to smartphone addiction instead of directing it toward the trouble source.

The study also found that anxiety and depression are positively related to executive dysfunction, thus supporting H2. The result is consistent with previous research (see reviews by Chen et al., 2014 and Wilmer et al., 2017). For example, Derakshan et al. (2009) found that the high-anxiety group showed poor performance in switching tasks compared with the control group. Levens and Gotlib (2010) used the n-back task to measure the updating of working memory, and the data revealed that patients with depression have difficulty in updating. Research conducted by Hartanto and Yang (2016) found that cognitive flexibility, working memory capacity, and inhibitory control are all impaired when anxiety is increased. In another study, Shields et al. (2016) induced anxiety in participants through self-reporting and found that the participants performed poorly on executive function tasks compared with the control group. The results can be explained as negative emotions exhausting cognitive resources and leading to executive dysfunction (Eysenck et al., 2007; Mitchell and Phillips, 2007).

Hypothesis H3 also was validated, and executive dysfunction is related to smartphone addiction based on previous research (Hadlington, 2015; Chen et al., 2016; Gao et al., 2020). Moreover, executive dysfunction is the mediation variable between anxiety and depression with smartphone addiction. H4 was supported. First, smartphone use has penetrated all aspects of our life. When in difficulties, we can depend on smartphones to seek information, to communicate with others, for entertainment, and for others as a coping strategy. Besides, anxiety and depression occupy cognitive resources, leading to executive dysfunction. In the process, executive dysfunction will cause failure to inhibit smartphone use and promote addiction formation. In summary, the role of executive dysfunction cannot be underestimated.

Moreover, it is worth noting that anxiety did not directly predict smartphone addiction and that executive dysfunction was completely mediating, contrary to previous findings (Elhai et al., 2020; Vally et al., 2021). We suspect that the result is due to two aspects. First, generalized anxiety may not directly increase smartphone addiction, and anxiety should be divided into more specific types, such as social anxiety (Przepiorka et al., 2021) and attachment anxiety (Han et al., 2017). In particular, smartphone addiction is associated with social anxiety (Ran et al., 2022). Second, the result fits attention control theory: smartphone addiction is the consequence of executive dysfunction promoted by anxiety-occupied cognitive resources, and executive dysfunction is the essential link that needs to be highlighted.

The study contributes to existing research on the underlying mechanisms of smartphone addiction formation. Executive dysfunction plays a mediated role between anxiety and depression with smartphone addiction. The finding supports I-PACE, IPM, and CIUT. In future studies, anxiety can be further subdivided into more specific types, such as attachment anxiety (Han et al., 2017; Liu et al., 2019), social anxiety (Hong et al., 2019; Kong et al., 2020), state anxiety (Shen et al., 2019), and academic anxiety (Yang Z. et al., 2019). In addition, except for executive dysfunction mentioned in the present study, more cognitive variables need to be explored between anxiety and depression with smartphone addiction.

Furthermore, it is essential to advocate using smartphones appropriately. The relationship between smartphone use and adaptive functioning is an inverse U-curve (Montag and Walla, 2016). Some researchers studied how to relieve smartphone addiction (Schmuck, 2020; Holte et al., 2021). Recently, 63 participants were asked to keep their smartphone screens in grayscale until their second visit. The result displays a significant decrease in smartphone addiction and anxiety levels (Holte et al., 2021). Another study found that digital detox apps (the apps used to monitor and limit smartphone use) may be useful for preventing the harmful effects of smartphone addiction (Schmuck, 2020).

This study has some limitations. The sample involves Chinese college students, which limits generalizability, and differences among different age groups and countries may exist. Furthermore, the purpose of usage and usage pattern of the smartphone are associated with smartphone addiction (Elhai et al., 2017b; Park, 2019), which needs further exploration. For instance, smartphone addicts are known to focus extensively on the social usage purpose of the smartphone (van Deursen et al., 2015). Furthermore, many people deny suffering from smartphone addiction (Park, 2019), and the measurement is done based on a lack of objectivity through self-reporting (Ryding and Kuss, 2020). Finally, Lim (2019) found that smartphone addiction predicts impaired executive function. Smartphone addiction may be involved in a vicious cycle of executive dysfunction. Our study is cross-sectional, and there are restrictions on examining causal relationships between factors, and more longitudinal studies are needed in the future.

In conclusion, we found that anxiety and depression are related to smartphone addiction in college students and that their link was mediated by executive dysfunction. Specifically, executive dysfunction completely mediated the pathway of anxiety and smartphone addiction and partly mediated the pathway of depression and smartphone addiction. Although the limitations of this study must be considered in future research, the tentative findings can inform research on treatment mechanisms of smartphone addiction and relieve the smartphone addiction from the source or the part.

## Data availability statement

The original contributions presented in the study are included in the article/supplementary material, further inquiries can be directed to the corresponding author.

## Ethics statement

The studies involving human participants were reviewed and the local research Ethics Committee of Chongqing Normal University approved this study and all participants signed informed consent. Written informed consent to participate in this study was provided by the participants' legal guardian/next of kin.

## Author contributions

JG conducted data collection and analysis, under the supervision of YL. All authors drafted the manuscript, provided critical revisions to the manuscript, and contributed to the conception of the work and research design.
